# *What to Look for in Relationships*: Development, inter-rater reliability, and initial validity estimates for a young child-caregiver relationship assessment

**DOI:** 10.3389/fpsyg.2023.1157665

**Published:** 2023-03-28

**Authors:** Joy D. Osofsky, Margaret A. Fields-Olivieri, Andrew L. Frazer, Rebecca A. Graham, Bethany H. McCurdy, Carl F. Weems

**Affiliations:** ^1^Louisiana State University Health Sciences Center, New Orleans, LA, United States; ^2^Human Development and Family Studies, Iowa State University, Ames, IA, United States

**Keywords:** child, observational, assessment, dyadic, caregiver

## Abstract

**Introduction:**

Observational assessments are important for understanding a range of behaviors and emotions in the young child-caregiver relationship. This paper provides initial data on a multidimensional assessment for professionals who work with young children and their caregivers, the *What to Look for in Relationships* (WLR). The WLR was designed to assist providers in evaluating strengths and areas for improvement in five areas of young child-caregiver relationship dimensions. This paper reports on the development, interrater reliability, initial convergent and discriminant validity, and incremental utility of the scales.

**Methods:**

Data were collected from caregiver-child dyads, who participated in a semi-structured observational caregiver-child interaction session as part of a clinic evaluation for relationship-based therapeutic services for young children in child protection. Recorded interactions were coded using the WLR scales with 146 interactions coded by at least two independent observers for interrater reliability analyses.

**Results:**

The scales showed adequate internal consistency, good inter-rater reliability, strong convergent associations with a single dimension measure (i.e., the Parent-Infant Relationship Global Assessment Scale; PIR-GAS) and discriminated those in the clinical range from those with adaptive functioning on the PIR-GAS.

**Discussion:**

This study provides initial support for the usefulness of the WLR scales for assessing dimensions of caregiver-child relationships during early childhood that may be useful targets of intervention.

## Introduction

Prevention and dyadic therapeutic services are crucial for at-risk infants, young children, and families. However, identifying behavioral or emotional difficulties in the first few years of life can be challenging before young children develop the capacity to use language to express feelings. Rather, young children often show how they feel through their behaviors and emotions. Increasing knowledge about brain development from neuroscience supports the critical role that early relationships play in influencing the development of the brain in both positive and negative directions (Center on the Developing Child, 2020). For young children, supportive relationships with adults, particularly those with primary caregivers, are crucial for physical development as well as healthy social and emotional development. In order to understand infant or young child psychological development, there is a need to consider their experiences over time within the context of meaningful relationships with parents and other caregivers (Osofsky, 2011; Osofsky et al., 2017).

For professionals from different disciplines working with infants and toddlers, including clinicians, home visitors, those doing early intervention, and early childcare and education providers, the focus needs to be placed on understanding infant and toddler emotional, social, and cognitive development. All of these competencies develop in the context of their caregiver relationships. It is also crucial that professionals working in this area understand that *behavior has meaning*. As Winnicott (1964; p. 88) so clearly articulated many years ago, “There is no such thing as a baby,” meaning that if you describe a baby, you are actually depicting a baby with someone else because the caregiver-infant relationship is crucial in understanding infant mental health and development. Infants and young children are exquisitely sensitive and dependent on their caregiving environment because they must exist and develop in the context of a caregiving relationship, be it a good one, or, in some cases, a poor relationship. In the following sections, some of the central positive and negative behaviorally observable indicators of the caregiver-child relationship that informed the development of the *What to Look for in Relationships* (WLR) interaction rating system are described.

Behavioral indicators of a positive caregiver-child relationship include the caregiver and child interacting and cooperating as well as displaying mutual positive affect. While the notion of caregiver-child engagement is operationalized in a variety of ways in existing literature, this conceptualization is similar to what Lindsey et al. (2010) have referred to as *parent-child mutuality*, which they operationalize as mutual compliance and shared positive affect. Similarly, Pasiak and Menna (2015) describe *interactional synchrony* in the parent-child relationship, which includes some of the descriptors of caregiver-child engagement in the present scale (e.g., mutual focus, exhibiting a high degree of reciprocity, and responsiveness to each other’s cues). Another similar construct is *facilitative play*, which Keren et al. (2005) describe as play that is creative, cooperative, and marked by positive affect. These constructs are indicators of a positive, healthy caregiver-child relationship and are consistently associated with positive child developmental outcomes such as emotional and behavioral self-regulation and social competence (Lindsey et al., 2009, 2010; Pasiak and Menna, 2015; Bornstein et al., 2018). Based on this, a mutual positive engagement scale was developed.

In addition to mutual engagement of the caregiver and child, caregiver behaviors that support the child’s learning and independent problem-solving are important indicators of a positive parent-child relationship. An important construct in this area is parent *scaffolding*, which is a joint problem-solving process in which caregivers structure tasks in accordance with the child’s abilities to support the child in completing tasks that they are not yet able to do independently (Fay-Stammbach et al., 2014; Mermelshtine, 2017). Scaffolding involves a caregiver providing assistance when a child is already working to accomplish a task and offering support and encouragement to promote learning rather than taking over for the child (Zurek et al., 2014). Central to scaffolding is caregiver sensitivity to the child’s developmental level and provision of age-appropriate assistance. In addition, parental use of encouragement and praise supports the child’s willingness to engage in problem-solving and learning activities, even if they are challenging. In addition, encouraging the child’s exploration of toys, following the child’s lead in play, and expanding on the child’s play themes are other important aspects of parenting that support the child’s agency and engagement, as well as the parent-child relationship (Dozier et al., 2018). Caregiver scaffolding and related behaviors are associated with other indicators of parent-child interaction quality, such as dyadic reciprocity, which encourages a child’s successful problem solving, persistence, and development of competence (Hustedt and Raver, 2002). These early positive behaviors can also help the child develop cognitive functioning related to school functioning and prosocial behavior (Fay-Stammbach et al., 2014; Mermelshtine, 2017).

This approach leads to outcomes including social engagement (Guttentag et al., 2014), peer competence (Lindsey et al., 2009, 2010; Pasiak and Menna, 2015), compliance, self-control, and peer acceptance (Pasiak and Menna, 2015). It is also related to increased capacity for the elaboration of play themes, level of organization, and verbal expressiveness (Keren et al., 2005); and better performance on spatial and numerical tasks (Sorariutta et al., 2017). Thus, a scale focusing on caregiver teaching, helping, and supporting development was developed.

On the negative side of parenting interactions, controlling or intrusive caregiver behaviors (e.g., being overly directive, physically intrusive) are indicators of poorer parent-child relationship quality (Creveling et al., 2010; Dehon and Weems, 2010). These behaviors are associated with less positive and synchronous caregiver-child interactions (Ispa et al., 2004) and may be related to the development of insecure or disorganized attachment styles (Cassidy et al., 2014). Controlling and intrusive parenting in early childhood is also associated with poor developmental outcomes, including lower intellectual functioning, poorer language and communication skills, poorer executive functioning, and poorer behavioral regulation (Hubbs-Tait et al., 2002; Pungello et al., 2009; Blair et al., 2011; Clincy and Mills-Koonce, 2013; Eisenberg et al., 2015). In addition, controlling and intrusive parenting is associated with higher child negativity and behavioral dysregulation and contributes to the development of externalizing behavior problems (Ispa et al., 2004; Stevenson and Crnic, 2013; Yan and Ansari, 2017).

A related sign of a potentially unhealthy caregiver-child relationship is when a parent seems unaware of the child’s developmental level or needs. This might include ignoring a child’s cues, directing play above or below the child’s developmental level, or providing the child too much or too little assistance. Such unresponsive or insensitive parenting is associated with a host of poor development outcomes, including insecure attachment styles, affect dysregulation, behavior problems, poor social competence, and later psychopathology (Leerkes et al., 2009; Leerkes, 2011; Lyons-Ruth et al., 2013). These behaviors may reflect a lack of knowledge of child development or awareness of the child’s developmental capacities (Bornstein et al., 2010). Not only do these caregiver behaviors denote poor interaction and relationship quality (Swanson et al., 2000; Ispa et al., 2004), they also may interfere with children’s healthy cognitive, language, self-regulatory, and emotional development (Hubbs-Tait et al., 2002; Pungello et al., 2009; Blair et al., 2011; Clincy and Mills-Koonce, 2013; Stevenson and Crnic, 2013; Eisenberg et al., 2015). Thus, we also created a set of items looking for caregiver intrusiveness and lack of awareness.

An extensive literature links caregiver negativity, hostility, and rejection with lower parent-child relationship quality (Newland et al., 2013) and risk for psychopathology and social difficulties later in development (Carson and Parke, 1996; Elam et al., 2014; Brooker et al., 2016; Moed et al., 2016; Norcross et al., 2017; Fang and Gagne, 2018). In particular, hostile and rejecting parenting is associated with the development of externalizing behavior problems and antisocial behavior (Carson and Parke, 1996; Shaw et al., 2000; Rubin et al., 2003; Trentacosta and Shaw, 2009; Harold et al., 2012; Slatcher and Trentacosta, 2012; Elam et al., 2014; Moed et al., 2016; Wagner et al., 2018). This may be because exposure to hostility and negativity from a caregiver may disrupt the development of healthy physiological (Brooker et al., 2016; Merwin et al., 2017) and emotional regulation patterns (Weems and Pina, 2010; Dagne and Snyder, 2011; Moed et al., 2016). Hostile and rejecting parenting is also associated with the development of internalizing problems, including both anxiety and depression (Brooker et al., 2016; Norcross et al., 2017; Fang and Gagne, 2018), as well as the development of poor social competence and social difficulties (Carson and Parke, 1996; Elam et al., 2014; Moed et al., 2016). Finally, hostile and rejecting parenting can also interfere with cognitive and executive function development (Blair et al., 2011; McFadden and Tamis-Lemonda, 2013). Thus, we also developed scale items for caregiver negativity.

Finally, while all young children exhibit negativity and non-compliance at times, certain negative behaviors, or child behavior that is predominantly negative during interactions with caregivers, may indicate poor caregiver-child relationship quality. For example, a child who rejects the parents’ attempts to join them in play or who maintains a physical distance from the caregiver may fear the caregiver or not know what to expect from their caregiver (Weatherston and Osofsky, 2009; Osofsky, 2018). When frequently occurring during the interaction, these behaviors may suggest an unsupportive or unhealthy caregiver-child relationship. In addition, challenging child behaviors can evoke negative or insensitive parenting responses. Child negative or oppositionality during play is associated with poor caregiver responsiveness, less parental structuring, and more intrusive caregiver behavior, as well as less interactional synchrony, positive affect, and playfulness between the parent and the child (Pasiak and Menna, 2015; Menashe-Grinberg and Atzaba-Poria, 2017). Thus, caregiver-child interactions and relationships are bidirectional (Funamoto and Rinaldi, 2014). Both caregiver and child contribute to the relationship, and therefore it is critical to capture both caregiver and child behaviors in assessments of caregiver-child relationships. Thus, a final set of items “looks for” child negativity.

Existing observational scales include two widely used observational assessments, the Parent-Infant Relationship Global Assessment Scale (PIR-GAS and the Dyadic Parent-Child Interaction Coding System (DPICS; Eyberg et al., 2013). The PIR-GAS was first introduced by Zero to Three (2005) in the Diagnostic Classification of Mental Health and Developmental Disorders of Infancy and Early Childhood Revised (DC: 0-3R). Using this diagnostic system, the PIR-GAS provides a single indicator of the quality of parent-infant relationships, with higher scores indicating higher relationship quality. The PIR-GAS has been found to be reliable (Müller et al., 2013) and has been used to differentiate families among general and clinical samples (including those with physical abuse histories; Hatzinikolaou et al., 2016). It has also been used as an outcome measure in clinical trials of caregiver-child interventions (e.g., Salomonsson and Sandell, 2011; Wright et al., 2018). The DPICS is most frequently employed as an assessment within Parent-Child Interaction Therapy (PCIT; Eyberg and Funderburk, 2011), which is an empirically-supported behavioral parenting intervention for children ages 2–7. While the PIR-GAS and the DPICS are widely used and demonstrate strong psychometric properties, they are limited in their ability to provide an understanding of the nuances of a caregiver-child relationship. Another widely used observational scale, the PICCOLO, was developed to understand interactions between parents and young children, 10–47 months. The PICCOLO measures 29 developmentally supportive parenting behaviors in four critical domains: affections, responsiveness, encouragement, and teaching (Roggman et al., 2013). While a helpful observational scale, the focus is more on observing parent behaviors than the interaction between parent and young child.

### The current study

Recognizing both the importance of observational assessment in evaluating early caregiver-child relationships as well as the inherent limitations in existing approaches, the *What to Look for in Relationships* (WLR) interaction rating system was developed to provide a comprehensive dyadic assessment of caregiver-child relationships across the five areas noted above. Drawing from our previous work and clinical experience (Weatherston and Osofsky, 2009; Osofsky, 2018), behaviors were identified to indicate that a relationship was going well and was supportive for the young child or that the relationship was not going well in terms of sensitivity to the young child’s needs. As described above, these behaviors centered upon Mutual Positive Engagement, Caregiver Helpfulness, Teaching and Support of Development, Caregiver Intrusiveness/Lack of Awareness, Caregiver Negativity, and Child Negativity toward Caregiver, which can be combined to create a Relationship Total Score. The interaction rating system was designed to easy to use and helpful for clinicians and also for a wide range of professionals including home visitors, early intervention professionals, and case workers with scoring being conducted while observing interactions.

We predicted that the features and behaviors could be reliably coded using interrater reliability estimates. We further predicted moderate internal consistency of the scale items and that the (WLR) would show convergent validity and incremental information beyond both a global assessment such as the PIR-GAS. We conducted initial discriminant validity analyses and report the mean score of those in the clinical range on the PIR-GAS.

## Materials and methods

### Participants

Data from the current study are from a record review of an existing clinical services program data set (more below). In this program, caregiver-child dyads were seen by clinicians at an academic medical center and were referred for services when there was a child coming into state custody in a local county who was under the age of six at the time of intake. Eighty-five children (57% male) participated in the data collection; however, some youth had multiple interactions recorded, such that some children were recorded interacting with more than one caregiver. Children were, on average, 35.05 months of age (SD = 21.63) when observed, but there was a wide range of ages. Specifically, 27 children (19.7%) were less than or equal to 12 months, 31 children (22.6%) were 13–24 months, 26 children (19.0%) were 25–36 months, 20 children (14.6%) were 37–48 months, 13 children (9.5%) were 49–60 months, and 20 children (14.6%) over 60 months. Caregivers observed included biological mothers (*n* = 43, 31.4%), biological fathers (*n* = 12, 8.8%), foster mothers (*n* = 60, 43.8%), and foster fathers (*n* = 22, 16.1%). Racial/ethnic, educational, and employment data was not collected by the program from foster parents; however, it was collected from biological parents. The sample of biological mothers was 78% African American, 16.2% White/Caucasian, and 5.4% “Other.” Over half (60.0%) of biological mothers had not completed high school, 31.5% had a high school diploma or a GED, and 8.6% had received a post-secondary certification or degree. The majority (81.1%) of biological mothers reported being unemployed. Mothers were on average 29.42 years old (SD = 5.51) at the time of the observation. Limited demographic information was available for only six of the biological fathers. Five identified as African American and one identified as White/Caucasian. Fathers were on average 37.82 years old (SD = 9.17) at the time of the observation. Because of the clinical program nature of the data set (i.e., children being seen with multiple caregivers) a total of 308 interactions were potentially available; however, only 146 interactions of the same dyad were coded by at least two independent observers for interrater reliability analyses and were then the focus of this analysis.

### Measures

The development of the *What to Look for in the Relationships* (WLR) observational interaction rating system was based on existing data and the authors’ clinical experience observing children and their parents or caregivers in many different situations—homes, child care centers, preschools, clinics, and primary care centers. As noted, items were developed by literature review, observations of caregiver-child interactions, and interviewing clinicians with expertise in infant mental health about their observations of caregiver-child relationships. The system has five subscales (coded with three options: 0, 1, or 2 observed, none, some or most of the time): Mutual Positive Engagement (5 items); Caregiver Helpfulness, Teaching, and Support (8 items); Caregiver Negativity (3 items), Caregiver Intrusiveness and Lack of Awareness of Child’s Developmental Needs (6 items), and Child Negativity Toward Caregiver (3 items). Negative Subscales are reverse coded such that higher scores indicate better quality of relationships (e.g., a high caregiver rejection score is indicative of low rejection). Subscale scores are tallied to yield a Relationship Total score. For the overall relationship score, higher scores are indicative of a more positive caregiver-child relationship. Copies are available upon request.

#### PIR-GAS

The Parent-Infant Relationship Global Assessment Scale (PIR-GAS) is a measure in the Diagnostic Classification of Mental Health and Developmental Disorders of Infancy and Early Childhood Revised (DC:0-3R), published by the Zero to Three Taskforce and designed to assess the quality of the parent-infant relationship. The PIR-GAS allows for a global rating of the quality of a parent-infant (or parent-child) relationship on a single continuous scale, with scores ranging from 1 to 100 and higher scores indicating higher relationship quality (Müller et al., 2013). In terms of clinical severity, scores lower than 81 are considered potentially indicative of clinical problems with scores between 1 and 40 labeled “Disordered,” 41–80 “Disturbed,” and scores 81–100 representing “Adapted” relationships. Previous research suggests good inter-rater reliability (*r* = 0.83, Aoki et al., 2002). PIR-GAS ratings in the current study were obtained from the individual clinicians treating cases and were also coded by independent coders with interrater reliability assessed as.89 (two-way mixed interclass correlation coefficients) in the current sample. This scale was used to estimate convergent validity with WLR scales and provide initial means and standard deviations for WLR scales among those in the clinical range on PIR-GAS (lower than 81).

### Procedures

As noted, data from the current study were collected with institutional IRB approval using record review of an existing clinical services program. For this research project, completely de-identified data were extracted from the program evaluation data set. The university Institutional Review Board (IRB) determined this study was “exempt” in accordance per federal regulations (45CFR46.102 and 21CFR56). In this program, caregiver-child dyads were seen by clinicians at an academic medical center in a large metropolitan Southern city. Families were referred for services if a child coming into state custody in a local county was under the age of six at the time of intake. The observational assessment was videotaped with consent from the parent and/or other custodial caregiver/legal guardian. Clinicians who observed and coded these interactions included psychology pre-doctoral interns, post-doctoral fellows, social workers, and psychologists as part of a multidisciplinary team evaluation. Caregiver-child dyads were observed participating in a semi-structured laboratory play session adapted for clinical purposes from the Crowell and Feldman (1988) research paradigm. The 85 children in this sample could have been observed more than once in the cases of more than one caregiver- leading to the 146 observations included for analysis - but the clinician coders for interrater reliability and subsequent analysis would be unique to each observation. The 45–60 min session was monitored through a one-way mirror and videotaped. The session included 10 min of free play, a 5 min clean-up, a series of four tasks graded by difficulty in which dyads worked at their own pace, and a 2 min separation followed by reunion.

Some modifications of the problem-solving session and tasks used were made to accommodate a wider age range and the clinic setting. Each child was given four tasks appropriate for their age and development. The third and fourth tasks exceeded the capabilities of the unassisted child. The caregiver was instructed to assist the child in any way he/she felt was needed. The graded tasks used readily available materials such as shape-boxes, puzzles, and blocks. No rewards was provided for successful completion of the tasks. The caregiver and child were alone in the room during the session. At the end of the interaction, a brief separation and reunion procedure was implemented, during which caregivers briefly left the child in the room alone and returned after approximately 2 min. The clinician observed from behind a one-way mirror and communicated with the caregiver by knocking on the door to indicate the appropriate time to transition to the next task. For reliability analyses, caregiver-child interactions were coded independently by two raters using the WLR.

## Results

To examine interrater reliability, two-way mixed (average measure, consistency) interclass correlation coefficients were calculated. Results indicated that the intra-class correlation (ICC) was.70 (95% confidence interval = 0.59–0.79) for Mutual Positive Engagement,0.74 (95% confidence interval = 0.63–0.81) for Caregiver Helpfulness, Teaching, Support of Development,0.62 (95% confidence interval = 0.48–0.73) for Caregiver Negativity,0.68 (95% confidence interval = 0.55–0.77) for Caregiver Intrusiveness/Lack of Awareness, and.81 (95% confidence interval = 0.74–0.87) for the Relationship Total Score. One of the scales indicated somewhat lower interrater reliability which was.59 (95% confidence interval = 0.43–0.70) for Child’s Negativity toward Caregiver.

Internal consistency was also calculated using both raters and for the total score and subscales. Estimates were as follows: Mutual Positive Engagement α = 0.83, Caregiver Helpfulness, Teaching, Support of Development α = 0.87, Caregiver Negativity α = 0.72, Caregiver Intrusiveness/Lack of Awareness α = 0.84, Child’s Negativity toward Caregiver α = 0.59, and Total Relationship α = 0.81.

Given that the child-caregiver interactions were observed among four different types of caregivers (biological mother *n* = 49, biological father *n* = 13, foster mother *n* = 62, foster father *n* = 22), we tested for variation in total relationship scores by type of caregiver with multilevel modeling analyses using HLM 8 (Raudenbush and Bryk, 2002). The HLM analyses nested the two raters’ Relationship total scores on Level 1 as a function of caregiver type on Level 2. Findings indicated a significant effect of caregiver type on Relationship total scores [t(143) = 10.79, *p* < 0.001]. To determine differences among caregiver types, we calculated the ICC for Relationship total scores by caregiver type. The ICC for biological mother was 0.80 (95% confidence interval = 0.64–0.89), 0.83 (95% confidence interval = 0.45–0.95) for biological father, 0.80 (95% confidence interval = 0.67–0.88) for foster mother, and 0.79 (95% confidence interval = 0.47–0.91) for foster father.

Means, standard deviations, skew, and score ranges are presented in Table 1. Overall, the descriptive analyses suggest adequate score distributions with a tendency for the total scale and subscales to be negatively skewed toward more positive relationships. Because of this we supplemented parametric Pearson correlations with the non-parametric Spearman’s rank order correlations.

**TABLE 1:** Correlation matrix, descriptive statistics, and summary of discriminant analyses.

	1	2	3	4	5	6	7	Mean (SD) for clinical range	Cohen’s *d*
1. Mutual positive engagement	–	0.64	0.43	0.54	0.42	0.82	0.76	6.10 (1.68)	−1.84
2. Caregiver helpfulness, teaching, support of development	0.71	–	0.46	0.69	0.37	0.89	0.76	10.24 (2.95)	−1.77
3. Caregiver negativity	0.52	0.61	–	0.5	0.20^†^	0.55	0.58	5.10 (1.01)	−1.27
4. Caregiver intrusiveness/lack of awareness	0.54	0.7	0.56	–	0.52	0.82	0.7	8.45 (2.16)	−1.62
5. Child’s negativity toward caregiver	0.42	0.39	0.25^†^	0.56	–	0.58	0.57	4.72 (0.94)	−1.08
6. Relationship total score	0.83	0.92	0.69	0.84	0.57	–	0.88	34.60 (6.61)	−2.23
7. PIR-GAS	0.8	0.79	0.66	0.66	0.51	0.86	–	N/A	–
Mean (SD)	7.80 (1.80)	12.94 (2.93)	5.63 (0.72)	10.21 (2.00)	5.25 (0.82)	41.90 (6.93)	83.40 (11.60)	–	–
Range	1–10	2–22	0–6	2–11	1–6	18–58	35–100	–	–
Skew	−1.04	−1.02	−2.67	−1.35	−0.87	−1.19	−1.31	–	–

Pearson are below the diagonal and Spearman Rank Order are above the diagonal.

^†^Indicates *p*-value significant at <0.05. All other *p*-values significant at <0.001.

To test convergent validity, Pearson correlations were conducted to assess associations among the identified Subscales with the PIR-GAS. We again took the average of the two raters for these analyses. The correlation matrix is presented in Table 1 and shows that each of the scales was moderately to strongly associated with the PIR-GAS scores. Spearman correlations are presented above the diagonal in Table 1 and show a similar pattern.

To test the incremental prediction by each of the subscales scores, a regression was calculated with each of the subscales regressed onto PIR-GAS scores. Results are summarized in Table 2 and shows that each subscale (with the exception of Caregiver Intrusiveness/Lack of Awareness) provides incremental prediction of PIR-GAS scores. Collinearity statistics suggest acceptable levels for incremental prediction.

**TABLE 2:** Regression results predicting Parent-Infant Relationship Global Assessment Scale (PIR-GAS) by *What to Look for in Relationships* (WLR) subscales.

Predictor	b	Std. error	b 95% CI (LL, UL)	t
(Intercept)	14.97	4.28	(6.50, 23.41)	3.50*
Mutual positive engagement	2.51	0.38	(1.76, 3.25)	6.67*
Caregiver helpfulness, teaching, support of development	1.16	0.27	(0.63, 1.69)	4.29*
Caregiver negativity	3.69	0.83	(2.05, 5.33)	4.45*
Caregiver intrusiveness/lack of awareness	0.13	0.37	(−0.60, 0.86)	0.35
Child’s negativity toward caregiver	2.24	0.69	(0.87, 3.61)	3.22*
Model R^2^ = 0.79* 95% CI (0.73, 0.85)	–	–	–	–

*Indicates *p* < 0.01.

Finally, we conducted a series of *t*-tests to determine if total relationship scores and subscale scores differentiated (discriminated) those in the clinical range on the PIR-GAS (i.e., those below 81)^1^. These results are summarized in Table 1 and show that the WLR total and subscales each discriminate those in the clinical range. Initial means for those who might be in the clinical range on the WLR are also provided in Table 1 and show that, in general, scores in the clinical range tended to be one standard deviation below the mean for the whole sample. Cohen’s d effect size estimates show large effect size differences from the clinical and non-clinical samples on each of the scales. Once again, given the skew, these were supplemented with non-parametric Mann–Whitney U tests, and identical patterns emerged.

## Discussion

The *What to Look for in Relationships* (WLR) interaction rating system was developed in an effort to systematically communicate the meaning of the behaviors and emotions observed in young children and parents or caregivers. Because there are multiple dimensions of relationship quality observational assessment is crucial in understanding early development, especially for young children who cannot express their feelings with language. The interaction rating system is helpful for clinicians and also may be useful for a wide range of professionals including developmentalists and other service providers such as home visitors, early intervention professionals, and case workers. An important strength of the WLR is that it not only provides an overall relationship quality score, like the PIR-GAS, but also specifies domain-specific subscales to identify specific relationship strengths and weaknesses. Results overall supported predictions that the features could be reliably coded using interrater reliability estimates, that the WLR would show convergent validity with the PIR-GAS, and that the scales would provide incremental information.

First, in terms of the overall total score, this may meet a general need by facilitating effective communication among providers with varying levels of training and expertise along a general domain. The overall scale had good interrater reliability, internal consistency, and convergent validity. However, the benefit, as noted, is the identification of specific areas where problems may exist. Parenting quality and caregiver-child relationship quality are not unidimensional constructs; different patterns of parent and child behaviors have distinct implications for child outcomes (McFadden and Tamis-Lemonda, 2013; Norcross et al., 2017). For example, intrusive parenting and rejecting or hostile parenting are often studied as one unitary negative parenting construct (McFadden and Tamis-Lemonda, 2013). However, it is possible for a parent to be high in one behavior and low in the other.

Parenting behaviors involving high levels of both intrusiveness and hostility are associated with the poorest cognitive outcomes, whereas parenting behaviors involving low hostility and high intrusiveness are associated with better cognitive outcomes (McFadden and Tamis-Lemonda, 2013). Moreover, poor or maladaptive parenting styles should be considered alongside parenting strengths and positive aspects of the parent-child relationship. In particular, although negative and intrusive discipline strategies are generally associated with increased risk for child aggression, if a parent is also responsive and supportive, this may protect the child from developing aggressive behaviors (Alink et al., 2012). Thus, the WLR scale not only provides guidance to service providers on whether a caregiver-child dyad requires a referral for further evaluation or treatment but also helps identify strengths and weaknesses in the relationship that may also indicate areas for future interventions.

An important strength of the WLR is that it was designed to be used by a variety of service providers with varying levels of clinical training and experience in observing and evaluating interactions between parents or caregivers and their young children. Although the DC:0-3R provides general guidance and a few examples to aid clinicians in making ratings, it provides few concrete, objective behaviors to guide behavioral observations of young children and caregivers. Often behavioral ratings are also done after carrying out a structured clinical interview with the parent. The DPICS, another clinical observation tool, does require that clinicians rate the frequency of objective, concrete behaviors. However, this measure also requires specific, extensive training and is therefore not accessible to many providers who may be interested in gaining more understanding of the caregiver-child relationship. Further, the PICCOLO, a widely used observation scale more on parent observations rather than the young child-parent interaction that is the main emphasis of the WLR interaction rating system.

While this study supports the validation of the WLR, it is not without limitations. First, the measure was created to provide more comprehensive insight into the child-parent relationship; however, it is impossible to capture all nuances. To address these concerns, open-ended questions were included regarding “Caregiver’s response to separation” and “What child does during separation” as well as “Other Comments” to capture additional strengths or concerns of the dyad. Scores on the WLR in this sample were skewed toward more positive relationships, highlighting the need to develop estimates in samples with larger portions of highly troubled relationships. On the other hand, the study was conducted using observations on a clinically-referred, high-risk sample. The results may differ with non-clinical samples where a ceiling effect of positive relationship behaviors may be seen. Replication and extension of the validation of the WLR is needed in non-clinical samples. The sample was also gathered from one location, which further limits the generalizability to larger populations. The cultural context of the infant and young child relationship is important to understand; the needs and behaviors of infants and toddlers may be interpreted differently depending on cultural values and expectations that can influence development (Osofsky et al., 2007; Osofsky and Lieberman, 2011; Osofsky, 2016; Thomas et al., 2019).

Families utilize childrearing practices that are consistent with their culture, beliefs, and the ways they were raised and often express these beliefs in different ways. As Roy Muir says so cogently in describing the clinical approach, *Watch, Wait, and Wonder*, “parenting comes naturally, but it comes naturally the way you learned it” (Muir, 1992; Cohen et al., 2006). Thus, it will be important to conduct future studies using the WLR behavioral rating system using larger samples and in multiple locations. Larger samples would also allow for the exploration of age, developmental, sociodemographic, and dyad effects. The WLR like most scales assumes that the greater the sensitivity between child and parent the better the interaction and the better the outcome. This approach is limited in assessing good enough interactions characterized by mismatches and repairs (Winnicott, 1964). The mismatch and repair requires multiple assessments to examine such changes in response. Future studies with the scale for clinicians and other professionals assessing relationships at multiple time points (both short term and longer term) will be helpful in learning more about its usefulness as are studies examining change after intervention to test sensitivity to change.

## Conclusion

Optimal child development occurs in the context of a healthy, supportive caregiver-child relationship; therefore, early identification of potentially at-risk or problematic caregiver-child relationships is critical (Osofsky, 2011; Osofsky et al., 2017). The *What to Look for in the Relationship Scale* was designed to be a user-friendly tool for a variety of service providers working with young children and their caregivers. The WLR asks providers to rate the occurrence of specific, concrete behaviors, which can then be summed to a single overall score or into subscales to identify specific relationship strengths and weaknesses. This scale provides an objective way to rate and report the behaviors and emotions being observed, and its use requires less training than other clinical tools like the PIR-GAS and the DPICS, as evidenced by the moderate degree of reliability between a clinician’s ratings and those of a research assistant with limited clinical training. The WLR may be particularly useful as a screening tool to identify families that may require a referral for additional services and relatively inexpensive. It gives the observer concrete information to include in a report to a school, to parents, or to juvenile court for young children involved in the child welfare system. Therefore, the *What to Look for in Relationships* scale objectifies the subjective reports of what is happening between the child and caregiver in order to be accessible to a wide range of service providers and to facilitate communication among them.

## Data availability statement

The raw data supporting the conclusions of this article will be made available by the authors, without undue reservation.

## Ethics statement

The studies involving human participants were reviewed and approved by Institutional Review Board, LSU Health Sciences Center, Department of Psychiatry. Written informed consent to participate in this study was provided by the participants’ legal guardian/next of kin.

## Author contributions

JO, MF-O, AF, and RG: conceptualization, data collection and curation, writing draft of manuscript, and editing. BM and CW: writing draft of manuscript, data analysis, conceptualization of data analysis, and editing. All authors contributed to the article and approved the submitted version.

## Footnotes

1 Only a very small proportion of interactions were below 40, thus we collapsed the two clinical groups on the Parent-Infant Relationship Global Assessment Scale (PIR-GAS).
